# The Value of Histopathological and Clinical Characteristics for the Assessment of the Prognosis and the Efficacy of Dynamic Anterior Stabilization Surgical Treatment for Shoulder Instability

**DOI:** 10.3390/diagnostics15243203

**Published:** 2025-12-15

**Authors:** Andrejs Finogejevs, Andris Jumtiņš, Eduards Toms Moritis, Sergejs Isajevs

**Affiliations:** 1 ORTO Klinika, 1A Bukultu Street, LV-1005 Riga, Latvia; 2 Department of Traumatology and Ortopaedics, Riga Stradins University, 16 Dzirciema Street, LV-1007 Riga, Latvia; 3 Landeskrankenhaus Villach, Nikolaigasse 43, A-9500 Villach, Austria; 4 Faculty of Medicine and Life Sciences, University of Latvia, LV-1004 Riga, Latvia; sergejs.isajevs@lu.lv; 5 Centre of Pathology, Riga East Clinical University Hospital, LV-1038 Riga, Latvia; 6 Centrala Laboratorija, 4A Gredu Street, LV-1019 Riga, Latvia

**Keywords:** dynamic anterior stabilization (DAS), clinical characteristics, histopathology

## Abstract

**Background:** Dynamic anterior stabilization (DAS) is a novel surgical technique for treating chronic anteroinferior glenohumeral instability. It presents an alternative to the currently used *Bankart* and *Latarjet* procedures, aiming to reduce associated complications and revision surgeries. However, the value of histopathological and clinical characteristics for the assessment of the prognosis and the efficacy of shoulder instability surgical treatment is still poorly understood. **Objectives:** The aim of this study was to evaluate the clinical effectiveness of DAS for anterior shoulder instability by analyzing clinical and histopathological characteristics. **Methods:** Twenty patients with anterior shoulder instability were included in the study. The patients underwent clinical assessments before the surgery and 1, 3, and 6 months after surgery. Tissue specimens from the anterior glenoid bone surface and a segment of the long head of the biceps tendon were evaluated after the surgery. **Results:** Our results demonstrated that the first three months postoperatively were characterized by worse functional outcomes; however, six months after the surgery, the patients demonstrated functional recovery. The extent of preoperative bone osteonecrosis was associated with functional outcomes after 6 months of surgical treatment (*p* = 0.034; χ^2^ = 0.482), whereas the extent of lymphocyte infiltration was associated with pain severity (*p* = 0.022; χ^2^ = 0.402). **Conclusions:** To conclude, our study showed that dynamic anterior stabilization is a clinically effective method, with functional recovery in 6 months. Furthermore, the associations of clinical and histopathological characteristics for the prognosis and assessment of anterior shoulder instability surgical treatment were observed.

## 1. Introduction

Anterior shoulder instability refers to a disruption of the soft tissue and/or bony structural integrity that results in the humeral head dislocating from the glenohumeral joint. It is a common condition, particularly affecting young males in their 20s, and the shoulder is the most frequently dislocated joint in the human body. The most common cause is trauma to the capsulolabral structures, although bony damage is also frequently observed. Clinically, the condition ranges from partial subluxation to complete dislocation, often accompanied by injury to the surrounding structures. Anterior dislocations account for approximately 95% of all shoulder dislocations [1,2,3].

Shoulder instability is commonly classified based on its cause, chronicity, and the direction of dislocation. Traumatic injuries, often abbreviated as TUBS (Trauma, Unilateral, Bankart lesion, Surgery), are the most prevalent and are typically precipitated by trauma, often resulting in structural damage. In contrast, atraumatic injuries, abbreviated as AMBRI (Atraumatic, Multidirectional, Bilateral, Rehabilitation first, Inferior capsular shift if surgery), generally occur in the absence of a single precipitating event. These injuries involve multiple microtraumas, leading to instability in multiple directions [3].

Currently, the two main surgical treatments are *Bankart* repair and the *Latarjet* procedure [3,4]. However, certain patient subgroups may benefit from a novel technique that combines elements of both. In this context, dynamic anterior stabilization may offer a promising solution.

A further distinction is made between first-time and recurrent dislocations. Shoulder stability is maintained by both static and dynamic stabilizers. Disruption of any of these components can predispose the joint to instability. The static stabilizers include the osseous anatomy, labrum, glenohumeral articulation, joint capsule, and negative intra-articular pressure [2,3].

Understanding the mechanisms behind shoulder instability requires a closer look at the anatomy. The shoulder is a complex anatomical unit composed of several joints—most importantly the glenohumeral joint—as well as capsuloligamentous and musculotendinous structures that work together to stabilize the shoulder while allowing movement across multiple planes [1,4].

The glenohumeral joint is a ball-and-socket, multiaxial joint formed by the articulation of the large humeral head with the shallow glenoid fossa of the scapula, which is encircled by the labrum. The glenoid fossa contacts only about one-third of the humeral head, enabling a wide range of motion in the axial, sagittal, coronal, and scapular planes. These movements are primarily driven by the rotator cuff and deltoid muscles, among others, which explains the shoulder’s inherent susceptibility to dislocation [1,2,3,4,5].

Shoulder instability implies varying degrees of damage to the static and/or dynamic stabilizers. The inferior glenohumeral ligament (IGHL) is one of the most critical structures in maintaining shoulder stability [1]. A *Bankart* lesion—defined as a detachment of the IGHL along with avulsion of the anteroinferior labrum from the glenoid—is a hallmark injury associated with anterior instability [5]. This lesion compromises both the labrum’s bumper-like function and the restraining force of the IGHL, particularly when the shoulder is in abduction and external rotation. As a result, the risk of anterior humeral head dislocation is significantly increased.

When the injury involves a fracture of the osseous glenoid rim, it is referred to as a bony Bankart lesion, which also occurs frequently. Additionally, an impaction fracture on the superolateral posterior aspect of the humeral head—caused by contact with the glenoid rim during dislocation—is known as a Hill–Sachs lesion [6].

Following a single dislocation event, the osseous, ligamentous, and labral structures may not heal completely, often resulting in residual laxity or deformity. With repeated dislocations, bone damage typically worsens, leading to progressively reduced joint stability. It is now well established that patients with chronic shoulder instability frequently exhibit both soft tissue and osseous defects—referred to as “bipolar” bone loss. This helps explain why soft tissue repair alone may fail in certain cases [7].

In current clinical practice, decision-making regarding treatment for shoulder instability places significant emphasis on the evaluation of bone loss. A glenoid rim defect involving more than 20% is generally considered an indication for bony reconstruction. Similarly, a severe Hill–Sachs lesion often necessitates a surgical approach that includes bone augmentation [3].

Glenohumeral instability is also a recognized risk factor for joint degeneration. Importantly, degenerative changes may occur even after stabilizing procedures, as these interventions can alter normal joint biomechanics and loading patterns [1].

The bone loss threshold for treating shoulder instability remains debated, but, generally, it is accepted that glenoid bone loss of more than 20–25% should be treated with bone augmentation methods, by the *Latarjet* procedure [1]. Complications after surgical treatment, reported in 5–15% of cases, can include neurovascular injury—especially to the musculocutaneous and axillary nerves—as well as graft-related issues like nonunion or malposition. Additionally, long-term radiographic osteoarthritis may develop in some patients 10–20 years after surgery [3,8].

Patients with anterior shoulder instability and no significant bone loss are typically treated with a Bankart repair. The goal of this procedure is to surgically reattach the detached labrum and associated ligamentous structures to restore the static stabilizing function of the anterior capsule. It is indicated in cases of Bankart lesions with minimal or no osseous involvement. Specifically, the glenoid bone loss should be below the critical threshold of 20%, and the Hill–Sachs lesion must be non-engaging [1,2,3].

Bankart repairs generally yield excellent clinical outcomes; however, the primary concern is the risk of recurrence or redislocation. Known risk factors for recurrence include younger age, male sex, participation in competitive collision sports, and unaddressed bone defects. One of the advantages of the Bankart repair is its low complication rate, as it does not involve osteotomy or hardware placement unlike the *Latarjet* procedure. The main long-term concern is the potential development of glenohumeral osteoarthritic changes [3,4].

Dynamic anterior stabilization (DAS) is a novel surgical technique for treating chronic anteroinferior glenohumeral instability. It presents an alternative to the currently used *Bankart* and *Latarjet* procedures, aiming to reduce associated complications and revision surgeries. DAS combines some advantages of bony transfers and soft-tissue augmentation procedures. DAS utilizes the long head of the biceps (LHB) tendon, transferring it through a subscapularis split to the anterior glenoid margin, creating a “sling effect” while simultaneously repairing the anterior labrum with a simple *Bankart* technique [8,9,10]. The method offers a dynamic and static stabilizing effect and can be performed in either an inlay or an onlay position, with both showing promising results. Onlay positioning may offer an additional labroplasty effect. However, long-term clinical studies are lacking [10]. While isolated Bankart repair presents a twofold greater risk of redislocation than *Latarjet* in cases with 10–20% glenoid bone lesions (GBLs), bony transfer procedures, such as *Latarjet,* for GBLs of 20–25% carry complication rates ranging from 16% to 30%. DAS may serve as an intermediate approach, avoiding the high recurrence rates of Bankart repair and the complications of Latarjet while addressing concomitant SLAP lesions [11].

The histopathological changes in capsulolabral structures in cases of traumatic injury include inflammatory changes in the tissue as well as degenerative and necrotic changes in the soft tissue and bone with hemorrhages in acute stages [12]. However, the morphological characteristics in bone and soft tissue in case of shoulder instability surgical treatment have been poorly understood. It could be suggested that the bone and soft tissue changes could be a significant factor influencing tissue remodeling and functional results after surgical treatment.

**The aim** of this study was to evaluate the clinical effectiveness of DAS for anterior shoulder instability by analyzing clinical and histopathological characteristics.

## 2. Materials and Methods

### 2.1. Study Design and Population

This was a single-center, prospective longitudinal cohort study.

**Figure 1:**
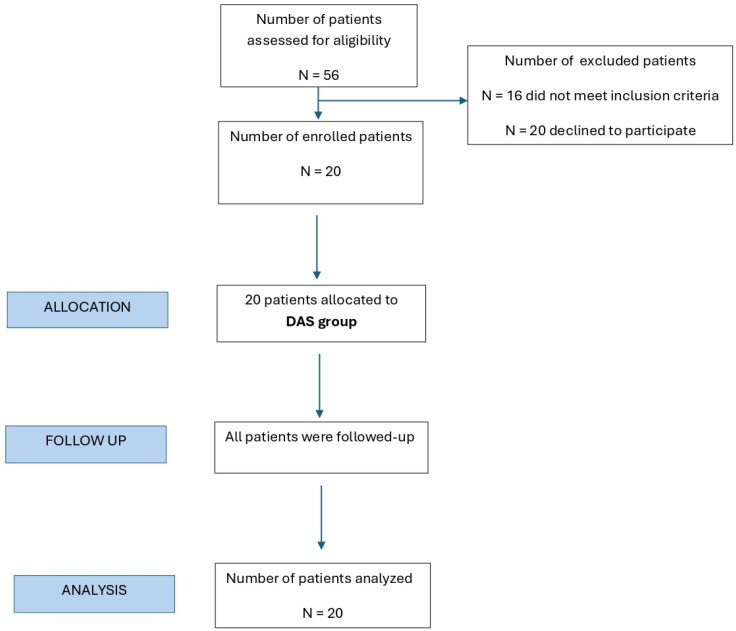
The flow chart of the study.

During outpatient visits, 56 patients with complaints of anterior shoulder joint instability were selected. Clinically, all patients had episodes of anterior shoulder joint instability. Subsequently, 16 patients were excluded from the study because they did not meet the inclusion criteria, whereas 20 patients refused to participate. Figure 1 demonstrates the flow chart of the study.

Twenty patients who underwent dynamic anterior stabilization (DAS) surgery were enrolled in the study. MRI confirmed soft-tissue injuries such as anterior capsulolabral complex damage, and CT of the shoulder joint confirmed a glenoid bone defect. According to clinical and radiological examinations, the patients did not have signs of osteoarthritis. All patients corresponded to the inclusions criteria and were treated between October 2023 and October 2025. The patient population consisted of 17 males and 3 females, with a mean age of 30.55 ± 7.65 years at the time of surgery.

During surgery, biopsy specimens were collected from each patient for histopathological analysis. Tissue samples were collected using a biopsy needle from the anterior glenoid bone surface before placing an intraosal anchor, and a segment of the long head of the biceps tendon was obtained.

All patients were followed-up using standardized clinical, imaging, and rehabilitation protocols. The presentation of this observational study corresponds to the Strengthening the Reporting of Observational studies in Epidemiology (STROBE) guidelines. In total, 22 checklist items were reviewed and consequently reported in the manuscript according to STROBE guidelines [13].

#### Inclusion and Exclusion Criteria

The inclusion criteria were as follows:

- Age under 50 years.
- History of anterior shoulder luxation or subluxation.
- Ongoing anterior shoulder instability.
- MRI-confirmed anterior labral lesion and Hill–Sachs lesion.
- CT-confirmed glenoid bone loss involving 10–20% of the total articulating surface.

The exclusion criteria were as follows:

- Age over 50 years.
- Presence of other structural damage within the shoulder joint.
- Multidirectional shoulder instability.
- Previous surgical intervention on the affected shoulder.
- Acute infection.
- Blood coagulation disorders.
- Systemic illnesses such as type 2 diabetes, rheumatoid arthritis, gout, or autoimmune diseases.
- Any missing clinical, histopathological, and imaging data.

### 2.2. Ethics Approval and Informed Consent

The study was conducted in accordance with the principles in the Declaration of Helsinki and the Oviedo Convention. Ethical approval was obtained from Riga Stradins University (No. 2-PEK–4/694/2024, 29 November 2024). The informed consent form was obtained from all participants in accordance with institutional requirements.

### 2.3. Clinical Evaluation, Follow-Up Schedule, and Rehabilitation

Clinical assessments were conducted at 1, 3, and 6 months. During each follow-up visit, patients were evaluated using the following standardized clinical outcome measures:

- Constant Shoulder Score (*CSS*).
- American Shoulder and Elbow Surgeons (*ASES*) Score.
- Oxford Shoulder Instability Score (*OSIS*).
- Visual Analog Scale (*VAS*).

In addition, the operative time and the need for additional analgesic medication were recorded.

All patients underwent the same rehabilitation protocol.

#### Histopathological Examination

The tissue specimens from the anterior glenoid bone surface and a segment of the long head of the biceps tendon were fixed in 10% neutral buffered formalin and embedded in paraffin. The core needle biopsy from the soft tissue (segment of the long head of the biceps tendon) of 8–12 mm length and from bone tissue (anterior glenoid bone surface) of 6–8 mm length was performed using a 07 G biopsy needle. The 3 μm thick sections were stained with hemotoxylin–eosin. The histopathological changes in bone and soft tissue were assessed. In the soft tissue the number of inflammatory cells (lymphocytes, leukocytes) were assessed in ten high-powered fields at magnification × 400, and the results were expressed as cells per mm/^2^.

Bone tissue histomorphometry was performed according to a 2012 update of the report of the ASBMR histomorphometry nomenclature committee [14]. The core length, tissue area, bone area, and bone volume were assessed. Osteonecrosis was defined as bone trabeculae demonstrating empty osteocyte lacunae. The results were expressed as the percentage of necrotic bone trabeculae.

Peritrabecular fibrosis was graded as follows: 0—regular bone trabeculae (distinct paratrabecular borders); 1—focal budding, hooks, spikes, or paratrabecular apposition of new bone; 2—diffuse paratrabecular new bone formation with thickening of trabeculae, occasionally with focal interconnections, 3—extensive interconnecting meshwork of new bone with overall effacement of marrow spaces.

### 2.4. Statistical Analysis

Categorical and non-parametric data were analyzed using the *Chi-square test* and the *Mann–Whitney U* test, respectively. Correlations between the clinical or histopathological variables were evaluated using the *Pearson Chi-square* (χ^2^) test. A *p*-value of <0.05 was considered statistically significant. The *GraphPad Prism* 12. version software was used for data analysis.

## 3. Results

In total, 20 patients were enrolled in the study. Table 1 demonstrates the clinical characteristics of the enrolled subjects: 17 patients were males, and 3 patients were females. The male/female ratio was 17/3. The median patient age was 30.55 ± 7.65 years.

Furthermore, 7 patients had left shoulder dislocation, whereas 13 patients had right shoulder dislocation.

The time from shoulder dislocation till surgery was 35 months (range, 2–97 months).

The median number of shoulder dislocations per patient was 6 (range, 2–9). The X-ray glenoid median defect was 13.90 ±3.08%.

**Figure 2:**
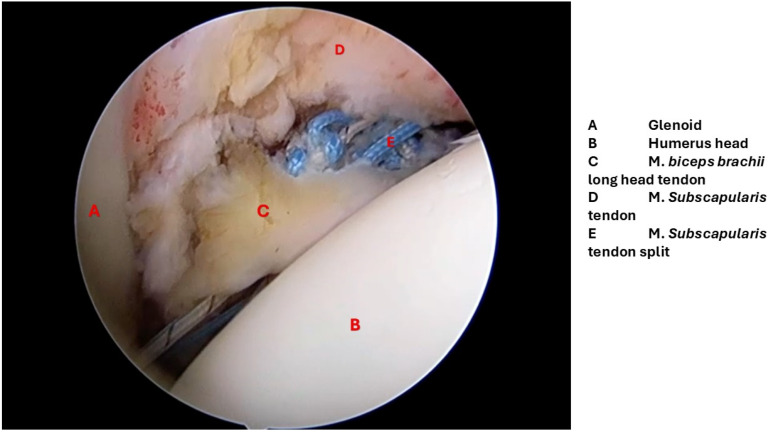
Representative arthroscopic microphotograph of dynamic anterior stabilization (DAS) surgery.

Figure 2 demonstrates a representative arthroscopic microphotograph of dynamic anterior stabilization.

**Figure 3:**
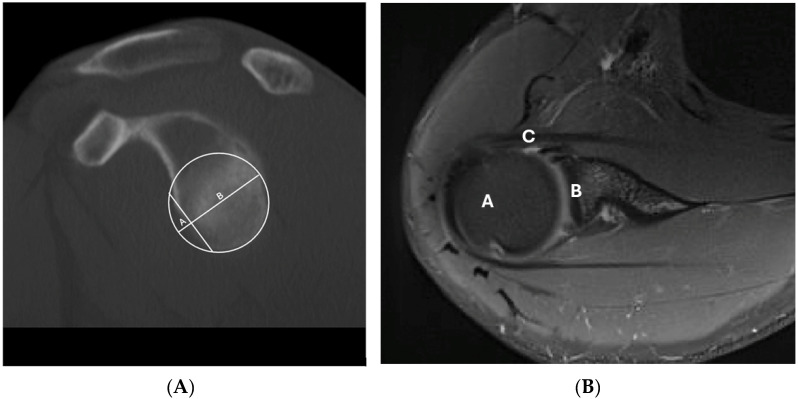
(**A**) Representative shoulder CT image demonstrating glenoid defect (sagittal bone); A—anterior bone defect of glenoid; B—glenoid bone. (**B**) Representative shoulder joint MRI image (pd_BLADE_fs_tra_256), A—humeral head, B—glenoid, C—labrum anterior lesion.

Figure 3A demonstrates a glenoid defect (sagittal bone) by CT imaging. Figure 3B shows a representative shoulder joint MRI image.

The Constant Shoulder Score (*CSS*), American Shoulder and Elbow Surgeons (*ASES*) score, Oxford Shoulder Instability Score (*OSIS*), and Visual Analog Scale (*VAS*) score were assessed before the surgery and 1, 3, and 6 months after the surgery.

The association of dislocation time to surgery with the *OSIS* was observed (*p* = 0.008; χ^2^ = 0.342). The numbers of dislocations was associated with shoulder dislocation (time)/reduction in hospital (*p* = 0.0087; χ^2^ = 0.480). The time of surgery was associated with the *ASES* score (*p* = 0.034; χ^2^ = 0.280). In addition, the *VAS* score was associated with the *CSS* (*p* = 0.03; χ^2^ = 0.360).

**Figure 4:**
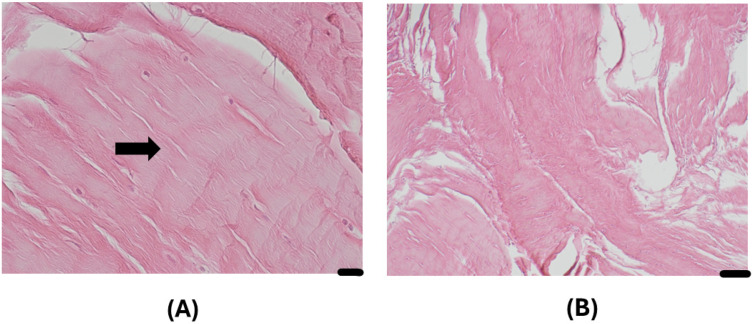
Histopathological changes in the segment of the long head of the biceps tendon. The long head of the biceps tendon tissue demonstrated reactive fibroconnective tissue with hyalinosis. The arrow indicates reactive fibroconnective tissue with hyalinosis. Hematoxylin–eosin staining method. (**A**) Magnification × 400, scale bar 20 µm. (**B**) Magnification × 100; scale bar—100 µm.

Histopathological examination from the segment of the long head of the biceps tendon after the surgery demonstrated mild-to-moderate reactive fibroconnective tissue with reactive myofibroblasts, hyalinosis, mild tissue edema, hemorrhage, and mild-to-moderate lymphocyte and leukocyte infiltration. Figure 4A,B demonstrate the reactive fibroconnective tissue with hyalinosis.

**Figure 5:**
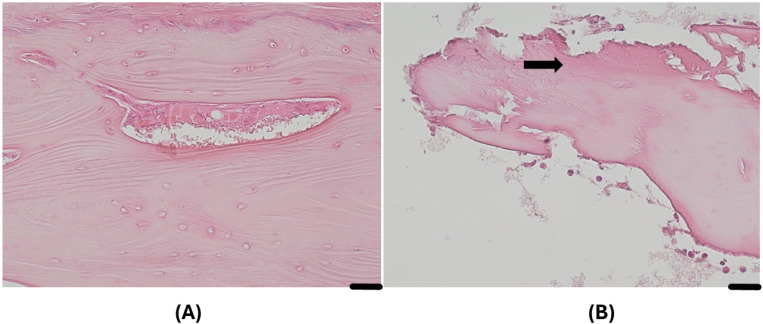
(**A**) Representative microphotograph of anterior glenoid cortical bone tissue. (**A**) Hematoxylin–eosin staining method. Magnification × 200, scale bar 50 µm. (**B**) Cortical bone trabeculae demonstrating empty osteocyte lacunae tissue (osteonecrosis). The arrow indicates osteonecrosis. Hematoxylin–eosin staining method. Magnification × 100, scale bar 20 µm.

Bone changes demonstrated fragmentation of bone trabeculae, hemorrhage, and mild leukocyte infiltration. Furthermore, in six patients, focal osteonecrosis was observed (Figure 5).

In addition, VAS score was associated with lymphocyte infiltration in the bone/soft tissue (*p* = 0.027; Rho = χ^2^ = 0.402).

The patients were followed-up for one, three, and six months. The *CSS*, *ASES* score, *OSIS,* and *VAS* were assessed at 1, 3, and 6 months.

**Figure 6:**
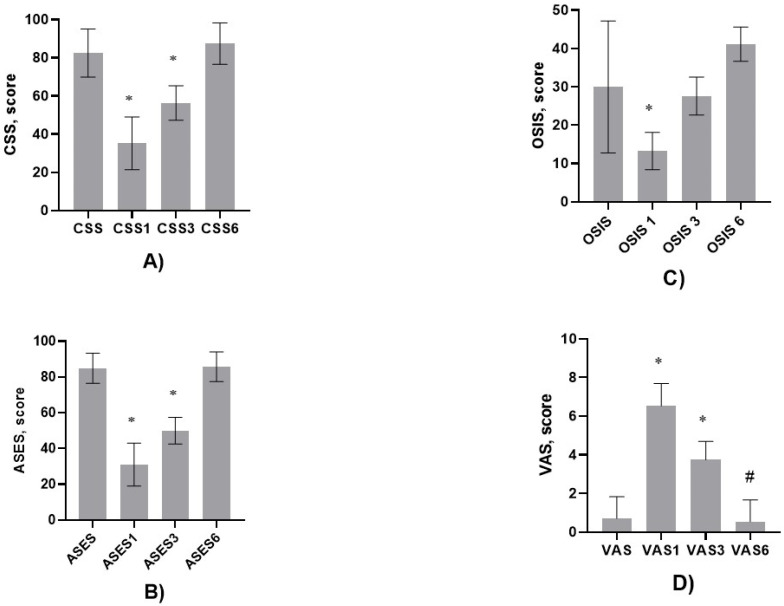
(**A**) The Constant Shoulder Score (*CSS*) before the surgery and 1 month, 3 months, and 6 months after the surgery. One-way ANOVA followed by Mann–Whitney U-test. * CSS vs. CSS1, *p* < 0.0001; * CSS vs. CSS3, *p* < 0.0001. (**B**) American Shoulder and Elbow Surgeons (*ASES*) score 1 month, 3 months, and 6 months after the surgery. One-way ANOVA followed by Mann–Whitney U-test. * ASES vs. ASES1, *p* < 0.0001; * ASES vs. ASES, *p* < 0.0001. (**C**) Oxford Shoulder Instability Score (*OSIS*) 1 month, 3 months, and 6 months after the surgery. One-way ANOVA followed by Mann–Whitney U-test. * OSIS vs. OSIS1, *p* < 0.0001. (**D**) Visual Analog Scale (*VAS*) 1 month, 3 months, and 6 months after the surgery. One-way ANOVA followed by Mann–Whitney U-test. * VAS vs. VAS1 and VAS vs. VAS3 *p* < 0.0001; ^#^ VAS1 vs. VAS6, *p* < 0.0001; ^#^ VAS3 vs. VAS6, *p* < 0.0001.

The obtained results showed that, one and three months after surgery, the *CSS* was significantly decreased compared with that before the operation, 35.21 ± 13.80 vs. 82.50 ± 12.61, *p* < 0.0001, and 56.33.00 ± 9.09 vs. 87.40 ± 10.89, *p* < 0.0001, respectively; Figure 6A.

However, after 6 months, the *CSS* did not differ from *CSS* values before the operation.

A similar trend was observed when the *ASES* score was analyzed. One and three months after surgery, the *ASES* score was significantly decreased compared with that before the operation, 31.00 ± 12.03 vs. 84.85 ± 8.41, *p* < 0.0001, and 50.00 ± 7.48 vs. 84.85 ± 8.41, *p* < 0.0001, respectively; Figure 6B.

However, after 6 months, the *ASES* score did not differ from the *ASES* score before the operation.

The *OSIS* one month after surgery decreased compared with that before the surgery, 13.26 ± 4.85 vs. 29.96 ± 17.22, *p* < 0.0001. However, it did not differ 3 months after surgery. In contrast, 6 months after surgery, the *OSIS* increased compared with the values before surgery: 41.13 ± 4.47 vs. 29.95 ± 17.22, *p* < 0.0001; Figure 6C.

The *VAS* score increased one and three months after the surgery, 6.53 ± 1.13 vs. 0.70 ± 1.13, *p* < 0.0001, and 3.73 ± 0.96 vs. 0.70 ± 1.13, *p* < 0.0001, respectively. However, the *VAS* score after 6 months significantly decreased compared with those at three and one month after and before the surgery (*p* < 0.0001); Figure 6D.

The obtained results showed that osteonecrosis was associated with the ASES score at 6 months, *p* = 0.044; χ^2^ = 0.482.

Correlative analysis showed that patient gender and age did not associate with ASES score, OSIS, and VAS score.

In addition, the number of dislocations was associated with CSS at 3 months after surgery (*p* = 0.0356; χ^2^ = 0.380). The shoulder dislocation (time)/reduction in hospital was associated with CSS and ASES score at 3 months after the operation, *p* = 0.039; χ^2^ = 0.275.

The X-ray glenoid defect was associated with the ASES score, *p* = 0.025; χ^2^ = 0.340.

## 4. Discussion

To the best of our knowledge, our study for the first time addresses the predictive value of histopathological and clinical characteristics 1, 3, and 6 months after anterior shoulder instability treatment by dynamic anterior stabilization (DAS).

Our results demonstrated that the first three months postoperatively were characterized by worse functional outcomes, reflecting the expected healing and rehabilitation period; however, six months after the DAS procedure, patients demonstrated non-inferior outcomes in the Constant–Murley Score (*CSS*) and the American Shoulder and Elbow Surgeons (*ASES*) score and improved outcomes in the Oxford Shoulder Instability Score (*OSIS*) compared with their preoperative status.

Shoulder pain, as measured by the Visual Analog Scale (*VAS*), also decreased after six months, both in comparison with preoperative values and with the 1–3-month postoperative period.

Our study showed that glenoid bone preoperative osteonecrosis, glenoid defect, the time between dislocation and surgery, and the number of dislocations were significantly associated with functional outcomes after surgery. These findings highlight the importance of early recognition and timely surgical management of shoulder instability, as well as careful patient selection. In addition, a higher number of lymphocytes in soft tissues was associated with higher *VAS* pain scores, suggesting that local inflammation may play a role in pain persistence and outcomes. Clinicians should keep these factors in mind, as they can affect functional recovery both in the short and long term.

Previous studies showed that DAS treatment is characterized by a lower complication rate. Clara de Campos Azevedo et al. [10] reported recurrence in only one patient (6.7%), while Philippe Collin et al. [9] found three recurrences (13.6%), mainly in earlier cases, reflecting the surgeon’s learning curve. Both studies, however, lacked a control group treated with alternative techniques by the same surgeon. Furthermore, previous studies have focused on retrospective and biomechanical analyses after surgical treatment. It has been demonstrated that DAS significantly reduced anterior glenohumeral translation compared with *Bankart* repair alone in shoulders with 10–20% GBLs [15,16]. A potential limitation was association with posterior and inferior humeral head shifts in abduction and external rotation (ABER). While the long-term effects remain unclear, posterior shifts greater than 5 mm have been linked to premature degenerative joint changes in cases with more-extensive bone loss [13]. Our results are consistent with previous findings and extend them by the observation of such an effect in a prospective study after 1-, 3-, and 6-month follow-up periods.

Previous studies showed that patient age and male gender were associated with a higher risk of surgery failure after primary shoulder stabilization [15]. In contrast, our study did not find an association between patient age and gender and surgery outcome. It could be suggested that the young age of the patients in our study (median age 28.5 years) could contribute to this.

Glenoid bone loss is well-known risk factor for failure of primary stabilization surgery [16,17,18]. Previous studies showed that combined glenoid and humeral head defects have an additive and negative effect on glenohumeral stability [19]. It has been shown that a threshold of 11% posterior glenoid bone loss implicated a 10 times higher surgical failure rate, while a threshold of 15% led to a 25 times higher surgical failure rate [20]. However, our study demonstrated that, following DAS surgical treatment, after 6 months of operation, the *CSS*, *ASES* score, *OSIS,* and VAS score significantly improved. This leads to the suggestion that DAS surgical treatment seems superior to other methods.

Osteonecrosis results in fibrous scar formation, with impaired clinical outcomes [17]. Our results are consistent with previous findings and extend them by demonstrating that the X-ray glenoid defect was associated with the *ASES* score.

The previous findings demonstrated that the changes in anterosuperior glenohumeral capsular ligament correlated with the shoulder trauma outcomes [21,22,23]. Furthermore, both dynamic and static factors contribute to the glenohumeral stability and the mobility of ligaments [24]. Our findings support and extend previous observations demonstrating that the extent of lymphocyte infiltration is significantly associated with the VAS score, contributing to the surgery outcomes.

Previous studies showed that the bone union started from 3 months postoperatively [25], which is consistent with our findings, which indicated that *CSS*, *ASES* score, *OSIS*, *VAS* value improved after 3 months but more significantly after 6 months.

From a clinical and biomechanical perspective, DAS offers several advantages. It allows the simultaneous treatment of SLAP lesions through LHB tenodesis and reproduces the three stabilizing mechanisms of the *Latarjet* (bumper effect, ligament reinforcement, and sling effect), while preserving the coracoid process and pectoralis muscle. This reduces neurological risk and scapular dyskinesis and strengthens *Bankart* repair in cases of poor-quality capsuloligamentous tissue [8,9]. Unlike *Latarjet*, *DAS* does not require screws or a full arthrotomy and is performed arthroscopically [9]. However, it is not suitable for GBLs > 20% or significant *Hill–Sachs* lesions unless combined with bone block or remplissage procedures [8]. Poor LHB tendon quality, previous biceps procedures, or capsular deficiency are additional contraindications, and axillary nerve injury during subscapularis perforation remains a theoretical concern [8].

DAS is primarily indicated for anterior instability with weakened capsular ligaments and GBLs ≤ 20% [8]. It is appropriate for SLAP type I–III lesions and positive apprehension tests in 90° abduction/external rotation [5,10]. Relative contraindications include GBLs of 20–30%, instability in overhead athletes without SLAP lesions, and subscapularis injuries [9]. Absolute contraindications include GBLs > 30%, prior LHB procedures, LHB rupture, and glenohumeral arthritis [5,9].

Compared with *Bankart* repair, *DAS* is more effective in cases of subcritical bone loss. *Bankart* repair alone remains suitable for minor lesions (<10%) but has higher recurrence rates in GBLs of 10–20%. The *Latarjet* procedure addresses larger bone defects effectively but is technically complex, requires pectoralis minor release, and carries a higher risk of neurological complications. *DAS* therefore offers an intermediate option, balancing efficacy with a lower complication profile [8,9,10,26,27,28,29,30]. In addition, the *ASES* score and the extent of glenoid defect have been shown as the major predictors of the outcomes of reverse total shoulder arthroplasty [31]. Furthermore, the ASES score has been found to be a major predictor in patients with recurrent anterior shoulder instability [32].

This study has several important strengths. First, it is a prospective clinical study evaluating the *DAS* procedure, whereas most of the existing literature is retrospective, clinical case descriptive, or biomechanical. This prospective design allows for a more reliable assessment of functional outcomes over time.

Second, the study provided comprehensive outcome evaluation, using both clinician-based subjective and objective scores (*CSS*, *ASES* score, *OSIS*, *VAS* score). This study allows us to evaluate not only the clinical objective side of DAS but also the changes in patients’ everyday life.

Third, different additional factors—such as glenoid osteonecrosis, defect size, time to surgery, and number of dislocations—can change the prognostic variables. These factors can help surgeons with future patient selection to improve outcomes in clinical practice.

Finally, the associations between clinical and histopathological characteristics shed light on personalized treatment and follow-up.

Despite these strengths, some limitations should be addressed. Firstly, the patient number was relatively small (20 patients undergoing DAS), which may decrease the statistical power to detect subtle differences between subgroups.

Secondly, the follow-up duration was limited to six months, which primarily captures short- to mid-term outcomes. Long-term data are required to determine the durability of DAS.

Finally, because DAS is a technically demanding arthroscopic procedure, results can vary based on the surgeon’s experience and learning curve. This factor should be considered when interpreting the complication and recurrence rates.

## 5. Conclusions

To conclude, our study showed the associations of clinical and histopathological characteristics for the prognosis and assessment of anterior shoulder instability surgical treatment. Histopathological characteristics like the extent of osteonecrosis and degree of lymphocyte infiltration were associated with the prognosis of functional recovery after surgical treatment. In addition, our prospective study demonstrated that the DAS procedure is a safe and effective option for treating chronic anterior shoulder instability with subcritical glenoid bone loss (10–20%).

## Figures and Tables

**Table 1:** Patients’ characteristics.

Characteristics	Value
Patients’ age, years	30.55 ± 7.65
Male/female ratio	17/3
First dislocation surgery (months)	35 (2–97)
Shoulder dislocation (time)/reduction in hospital	4 (range 2–12)
Dislocations, median (range), n	6 (2–9)
Surgery after last dislocation, months	35 (2–97)
X-ray, glenoid defect, %	13.90 ± 3.08
Soft tissue, leukocyte score	1 (0–2)
Soft tissue, lymphocyte score	1 (0–2)
Tissue area, mm^2^	34 ± 6.24
Bone area, mm^2^	22.6 ± 8.12
Bone volume, %	52.8 ± 17.4
Peritrabecular fibrosis	1 (1–2)
Osteonecrosis, %	6.35 ± 7.88

## Data Availability

The original contributions presented in the study are included in the article, further inquiries can be directed to the corresponding author.
